# RNA Sequencing Reveals Candidate Genes and Pathways Associated with Resistance to MDM2 Antagonist Idasanutlin in *TP53* Wild-Type Chronic Lymphocytic Leukemia

**DOI:** 10.3390/biomedicines12071388

**Published:** 2024-06-22

**Authors:** Erhan Aptullahoglu, Sirintra Nakjang, Jonathan P. Wallis, Helen Marr, Scott Marshall, Elaine Willmore, John Lunec

**Affiliations:** 1 Biosciences Instittute & Newcastle University Cancer Centre, Medical Faculty, Newcastle University, Newcastle upon Tyne NE2 4HH, UK; elaine.willmore@ncl.ac.uk; 2 Department of Molecular Biology and Genetics, Faculty of Science, Bilecik Şeyh Edebali University, 11100 Bilecik, Türkiye; 3 School of Medicine, Dentistry and Biomedical Sciences, Queen’s University Belfast, Belfast BT7 1NN, UK; s.nakjang@qub.ac.uk; 4 Department of Haematology, Freeman Hospital, Newcastle upon Tyne NHS Foundation Trust, Newcastle upon Tyne NE7 7DN, UK; jpwallis@btinternet.com (J.P.W.); helen.marr@nuth.nhs.uk (H.M.); 5 Department of Haematology, City Hospitals Sunderland NHS Trust, Sunderland SR4 7TP, UK; scott.marshall@chsft.nhs.uk

**Keywords:** RNA sequencing (RNA-seq), MDM2–p53 antagonists, idasanutlin (RG7388), DNA damage repair, chronic lymphocytic leukemia (CLL)

## Abstract

Chronic lymphocytic leukemia (CLL) is a genetically and clinically diverse hematological cancer affecting middle-aged and elderly individuals. Novel targeted therapy options are needed for patients who relapse following initial responses or who are intrinsically resistant to current treatments. There is a growing body of investigation currently underway on MDM2 inhibitors in clinical trials, reflecting the increasing interest in including these drugs in cancer treatment regimens. One of the developed compounds, idasanutlin (RG7388), has shown promise in early-stage clinical trials. It is a second-generation MDM2–p53-binding antagonist with enhanced potency, selectivity, and bioavailability. In addition to the *TP53* status, which is an important determinant of the response, we have shown in our previous studies that the *SF3B1* mutational status is also an independent predictive biomarker of the ex vivo CLL patient sample treatment response to RG7388. The objective of this study was to identify novel biomarkers associated with resistance to RG7388. Gene set enrichment analysis of differentially expressed genes (DEGs) between RG7388-sensitive and -resistant CLL samples showed that the increased p53 activity led to upregulation of pro-apoptosis pathway genes while DNA damage response pathway genes were additionally upregulated in resistant samples. Furthermore, differential expression of certain genes was detected, which could serve as the backbone for novel combination treatment approaches. This research provides preclinical data to guide the exploration of drug combination strategies with MDM2 inhibitors, leading to future clinical trials and associated biomarkers that may improve outcomes for CLL patients.

## 1. Introduction

Next-generation RNA sequencing (RNA-seq) can be used to quantify the simultaneous genome-wide expression of thousands of genes at great depths. It is a useful approach for investigating complex transcriptomic changes, e.g., measurement of differentially expressed genes between two conditions such as treated vs. non-treated cells or between tumor samples from two different subgroups of patients [1].

We have previously reported the concentration-dependent cytotoxic effect of the second-generation small-molecule inhibitor of the p53–MDM2 interaction, idasanutlin (RG7388), on ex vivo-tested TP53^WT^ primary chronic lymphocytic leukemia (CLL) patient samples, when compared with normal hematopoietic and bone marrow cells, and TP53^MUT^ CLL cells [2]. RG7388 was the first MDM2–p53-binding antagonist to progress through phase II clinical studies (ClinicalTrials.gov identifier: NCT02633059). According to preliminary clinical trial outcomes, RG7388 offers potential as a therapeutic drug for leukemia [3]. However, the CLL cell response to MDM2 inhibitors varies considerably, and although dependent on the *TP53* mutational status, it also shows variation across *TP53*^WT^ samples. *SF3B1* mutations in CLL patient samples were found to be associated with resistance to treatment with RG7388 ex vivo, and patients with the wild type for both *SF3B1* and *TP53* are most likely to benefit from treatment with MDM2 inhibitors [4]. To further explore the mechanisms involved in the differences in drug responses, RNA-seq analysis was carried out on a selection of TP53^WT^ primary CLL samples either resistant or sensitive to the MDM2 inhibitor RG7388, to define differentially expressed genes and pathways activated by RG7388 compared to untreated controls. The main objective in this study was to identify potential response biomarker signatures as well as possible novel targets for combination treatments.

## 2. Materials and Methods

### 2.1. Patient Samples

Peripheral blood samples were obtained from CLL patients with informed consent, in accordance with institutional guidelines and the Declaration of Helsinki. CLL patient samples were obtained and stored under the auspices of the Newcastle Biobank (Research Ethics Committee (REC) reference 17/NE/0361). CLL diagnosis was made according to IWCLL-164 NCI 2008 criteria [5]. CLL patient samples were cultured using RPMI-1640 medium (Sigma-Aldrich, St. Louis, MO, USA) with 10% fetal calf serum and 100 U/mL penicillin/streptomycin (Sigma-Aldrich, St. Louis, MO, USA). Idasanutlin (RG7388) was dissolved in DMSO (Sigma-Aldrich) and used at a final concentration of 0.5% DMSO (*v*/*v*). The compound was custom synthesized with >99% purity as part of the Newcastle University/Astex Pharmaceuticals Alliance and CRUK Drug Discovery Programme at the Newcastle University Cancer Centre.

### 2.2. Patient Sample Information

The details of Sanger sequencing of *SF3B1* in primary CLL samples have been described in a previous study [6]. The *TP53* mutational status of CLL samples was assessed by next-generation sequencing (using Roche 454 GS FLX and Illumina MiSeq platforms) in all samples. The presence of a 17p deletion was assessed by fluorescence in situ hybridization and/or multiplex ligation-dependent probe amplification analysis. The functional status of p53 in CLL samples was determined by observing the stabilization of p53 and activation of downstream protein, MDM2, following short-term exposure to the MDM2 inhibitor RG7388. Viability (routinely assessed by a trypan blue exclusion assay) was >95% in fresh and thawed samples after a 24 h culture.

### 2.3. Cell Viability Assay

We exposed 5 × 10^6^ cells/mL in 100 μL of medium per well of a 96-well to a range of concentrations (from 1 to 10^4^ nM) of idasanutlin for 48 h. The ex vivo cytotoxicity was assessed by using an XTT Assay Kit II (Sigma-Aldrich, Gillingham, UK). The results were normalized to DMSO controls and expressed as % viability.

### 2.4. RNA Extraction

RNA-seq was carried out on thawed CLL primary samples. As more than 90% of PBMCs in a CLL patient’s peripheral blood were found to be CD5+ CD19+ B cells (based on previous studies in our group performed with CLL primary cells and personal communication with clinical staff), no further isolation was carried out on CLL cells. In addition, peripheral-blood samples were obtained from CLL patients with total white blood cell counts of at least 30 × 10^9^/L, supporting the high proportion of malignant B cells. PBMCs were transferred from the cryogenic vial to pre-warmed RPMI-1640 media supplemented with final concentrations of 10% (*v*/*v*) fetal calf serum (FCS), 100 U/mL penicillin, 100 µg/mL streptomycin, and 2 mM L-glutamine, and washed twice in 5 mL pre-warmed media. The cell pellet was then resuspended in 1 mL pre-warmed media. After counting the cells and checking the viability with the trypan blue exclusion test, cells were seeded in 6-well plates (Corning, Corning, NY, USA) at 5 × 10^6^ cells/mL and 2 mL/well. After incubating in a 37 °C and 5% CO_2_ incubator for one hour, cells were exposed to 1 µM RG7388 for 6 h at a final DMSO solvent concentration of 0.5% (*v*/*v*). Solvent (DMSO-alone) controls were also included for each individual patient sample. Treatment of cells with 1 µM RG7388 for 6 h had been shown in previous experiments to activate the expression of several p53 pathway genes with minimal cytotoxic effects on CLL cells at this dose and time point [2]. At the time of the harvest, the medium was removed and cells were washed once with phosphate-buffered saline (PBS), then collected in microcentrifuge tubes. Total RNA was extracted from the pellets using an RNeasy Mini Kit (Qiagen, Hilden, Germany) according to the manufacturer’s protocol. A DNAse treatment step with on-column digestion was carried out using the RNase-free DNase kit (Qiagen). The RNA purity and concentration were determined by measuring the optical density (O.D.) at 260 nm with an ND-1000 spectrophotometer (Thermo Fisher Scientific, Horsham, UK). The A_260_/A_280_ ratio was used to assess RNA purity. An A_260_/A_280_ ratio of 1.8–2.1 is indicative of highly purified RNA.

### 2.5. RNA Quality *Assessment*

The RNA integrity was also evaluated using an Agilent 2100 Bioanalyser before RNA-seq. Agilent 2100 Expert Software (B.02.08 version) uses an algorithm that takes characteristics of several regions of the recorded electropherogram into account to calculate an RIN (RNA Integrity Number) value from a small amount of initial RNA, and it offers a user-independent tool for standardization of RNA quality control. RIN values range from 1 (worst) to 10 (best), with values above 8 being acceptable for most applications [7]. The concentration of mRNA samples was determined and RIN value quality assessment performed using an Agilent RNA 6000 Nano kit on an Agilent 2100 Bioanalyser before RNA-seq (Supplementary Figure S1 and Supplementary Table S3). An RNA 6000 Ladder (Thermo Fisher Scientific, #AM7152) was used with Agilent’s lab-on-a-chip system for size estimation.

### 2.6. RNA-Seq

Sequencing was performed by the Genomics Core Facility at Newcastle University, UK, on a NovaSeq 6000 Sequencing System (Illumina, Cambridge, UK) at 33 million reads per sample (minimum read length 2 × 75 bp), using the Illumina TruSeq^®^ Stranded mRNA Sample Preparation Kit and following the manufacturer’s instructions. The first step in the workflow involves purifying the poly-A containing mRNA molecules using poly-T oligo-attached magnetic beads. Following purification, the mRNA is fragmented into small pieces and copied into first-strand cDNA using reverse transcriptase and random primers. To synthesize the second cDNA strand, dUTP instead of deoxythymidine triphosphate (dTTP) was used. The incorporation of dUTP in second-strand synthesis quenches the second strand during amplification, because the polymerase used in the assay does not combine with this nucleotide. Magnetic beads separate the ds cDNA from the second-strand reaction mix. At the end of this process, blunt-ended cDNA is obtained. In this step, actinomycin D was used to increase strand specificity by inhibiting second-strand cDNA synthesis. These cDNA fragments then have the addition of a single ‘A’ base and subsequent ligation of the adapter. The products are then purified and amplified by PCR to create the final cDNA library.

The initial analysis and quality control checks were performed on the raw sequencing data. We aimed to address questions regarding gene expression, including 1. Assessment of the differential gene expression in untreated vs. 1 µM RG7388 treated samples, and 2. Determination of the presence of differentially expressed genes in RG7388-sensitive vs. -resistant patient subgroups.

### 2.7. Real-Time Reverse Transcriptase Polymerase Chain Reaction Gene Expression Analysis

We seeded 5 × 10^6^ cells/mL in 2 mL per well of a 12-well plate and exposed them to 1 µM of RG7388 or DMSO for 6 h. Total RNA was isolated using an RNeasy Mini Kit (Qiagen, Hilden, Germany). Complementary DNA (cDNA) was generated using the High-Capacity cDNA Reverse Transcription Kit (Applied Biosystems by Thermo Fisher Scientific, Horsham, UK, #4368814) as described by the manufacturer. qRT-PCR was carried out using SYBR green RT-PCR master mix (Life Technologies by Thermo Fisher Scientific, Horsham, UK) as per the manufacturer’s guidelines using the gene-specific cDNA primers shown in Table 1. An Applied Biosystems QuantStudio™ 7 Real-Time PCR System (Applied Biosystems by Thermo Fisher Scientific, Horsham, UK) was used to run PCR reactions. Analysis was carried out using SDS 2.2 software (Applied Biosystems). Each sample was analyzed in triplicate using GAPDH as a housekeeping control. A no-template control was used to control for contamination of external DNA in the reactions. The mRNA expression of each gene, expressed as Ct values (cycle number to reach critical threshold), was compared with its DMSO-treated matched sample.

### 2.8. Immunoblotting

We seeded 5 × 10^6^ cells/mL primary CLL cells in 2 mL per well of a 6-well plate (Corning) and subjected them to treatment with idasanutlin. Protein lysates were harvested using 2% SDS lysis buffer at 24 h, heated at 95 °C for 10 min, and sonicated. The protein concentration was measured using a Pierce™ BCA Protein Assay Kit (Thermo Fisher Scientific, Horsham, UK). Primary antibodies against p53 (DO-7) (#M7001, Dako), MDM2 (Ab-1) (#OP46, Merck Millipore, Burlington, MA, USA), PUMA (#PC686, Calbiochem, San Diego, CA, USA), and actin (Sigma) and secondary goat anti-mouse/rabbit horseradish peroxidase-conjugated antibodies (Dako, Santa Clara, CA, USA) were used. All antibodies were diluted in 5% (*w*/*v*) nonfat milk or BSA in TBS-tween20. Proteins were visualized using enhanced chemiluminescence reagents (GE Healthcare, Amersham, UK).

### 2.9. Statistical Analysis

Statistical tests were carried out using GraphPad Prism 6 software.

## 3. Results

### 3.1. Selecting TP53 Wild-Type Differentially Responsive Patient Samples for Transcriptome Analysis

**Figure 1:**
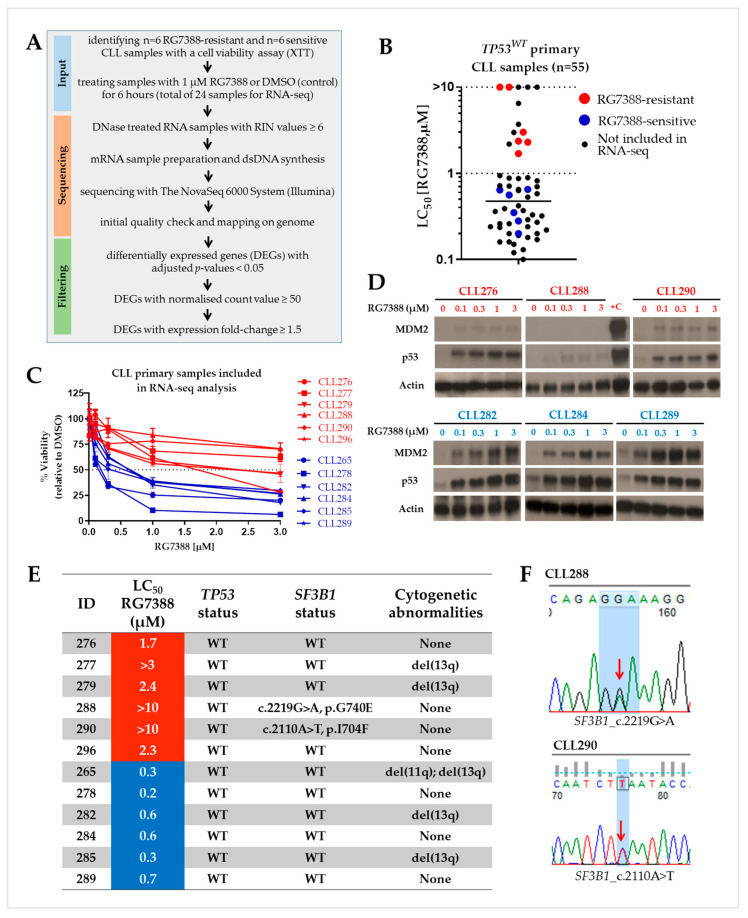
Defining sub-populations and differential response to RG7388, and some important features of the CLL samples selected for the RNA-seq study. (**A**). Workflow of the RNA-seq data analysis. (**B**). LC_50_ values showing the range of responses to RG7388 in fifty-five *TP53*^WT^ primary CLL samples. RG7388-resistant (red; *n* = 6) and -sensitive (blue; *n* = 6) samples selected for the RNA-seq analysis are marked with different colors. (**C**). Cytotoxicity curves for RG7388-resistant (red) and RG7388-sensitive (blue) CLL samples included in RNA-seq analysis. Samples were exposed to increasing concentrations of RG7388 (from 0 to 10 µM) for 48 h. All chosen samples were wild-type for the *TP53* gene. Cell viability was assessed by an XTT assay. (**D**). Western blots of three RG7388-resistant (red) CLL samples showed low levels of p53 stabilization saturated at the lowest drug concentration with little or no activation of the downstream protein MDM2. Concentration-dependent stabilization of p53 and the activation of the downstream protein MDM2 were clearly evident in the RG7388-sensitive (blue) samples. Actin was used as a loading control. +C: positive control lysate from NGP cells treated with 2.5 µM Nutlin-3a for 4 h. (**E**). Some important genetic features that may affect the response to RG7388. The *TP53* mutational status was assessed by targeted next-generation sequencing. Sanger sequencing was used to detect *SF3B1* mutations. WT: wild type. (**F**). Sanger sequencing showing CLL288 and CLL290 have heterozygous point missense mutations in the *SF3B1* gene. Red arrows show the point mutations.

The *TP53* and *SF3B1* gene statuses were previously found to be statistically significant independent predictive variables for the response of CLL patient samples to RG7388 [2,4]. The *TP53* status specifically distinguishes a subgroup of intermediate responders (1 µM < LC_50_ < 10 µM) and resistant responders (LC_50_ ≥ 10 µM) from the sensitive subgroup (LC_50_ ≤ 1 µM). We have previously shown that the *TP53* status in the subgroup of patients who are moderately responsive to RG7388 differs from the drug-sensitive group, and the *TP53* mutant allele frequency also has an effect on the variable response [2]. However, the resistance mechanism in some *TP53* wild-type subpopulations that are expected to respond to RG7388 is still not fully explained. The *SF3B1* mutational status is an another important predicter in CLL [4], but other response biomarkers need to be investigated in CLL cells that have neither *TP53* nor *SF3B1* mutations but are still resistant. We, therefore, planned an RNA-seq study for a large-scale transcriptome analysis. The criteria we used in sample selection and the characteristics of the samples are shown in Figure 1. RNA-seq was carried out on a total of twelve CLL primary samples untreated (DMSO control) or treated with a standard dose of 1 µM RG7388 for 6 h. Treatment of cells with a standard dose of 1 µM RG7388 for 6 h had been shown in our experiments to activate early expression of several p53 pathway genes with minimal cytotoxic effects on CLL cells at this dose and time point [2].

### 3.2. Principal Component Analysis (PCA) and Initial Assessment of Differentially Regulated Genes

**Figure 2:**
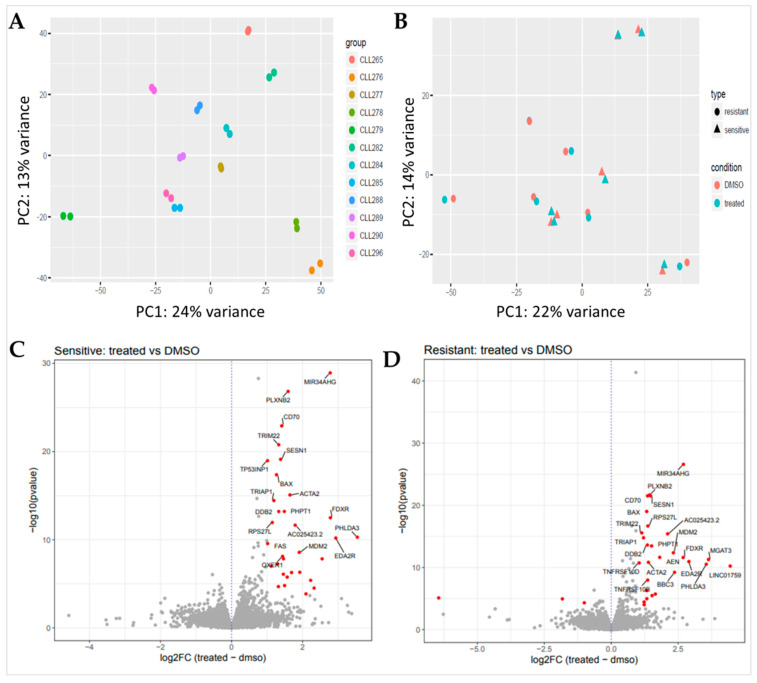
Principal component analysis (PCA) and volcano plot of differentially regulated transcripts. (**A**). PCA plot of RNA-seq data show the characteristics of samples according to gene expression levels. Each dot indicates an individual sample; the same color indicates samples from the same individual, whether treated or untreated with 1 µM RG7388 for 6 h. The score plots display each sample in the dataset with respect to the first two principal components and can therefore be used to interpret the relations among the samples. This information can be used to identify outliers. The samples belonging to the same CLL patient (either treated or untreated) show a high similarity with respect to the first two principal components, and a much greater variance between samples. (**B**). There was no clear separation into RG7388-resistant and -sensitive groups for either control (DMSO) or treated patient samples. (**C**,**D**). Volcano plots of altered gene expression profiles for RG7388-sensitive (**C**) and -resistant (**D**) CLL samples after drug treatment. A Benjamini–Hochberg-corrected significance value of <0.05 was used as a cut-off. The red dots represent significantly up- or downregulated genes (|log2 FC| ≥ 1 and FDR < 0.05), and the gray dots represent non-significant gene expression changes (|log2 FC| < 1 and/or FDR ≥ 0.05). FC: fold change; FDR: false discovery rate.

A principal component analysis (PCA) plot provides insights into the degree of similarity between mRNA expression patterns in samples [9]. The PCA plot showed closely similar expression profiles between untreated (DMSO control) and treated samples from the same individual. There was no clear similarity grouping between individual patient samples (Figure 2A). In particular, there was no clear grouping into resistant and sensitive samples detected (Figure 2B). Interestingly, the difference in profiles between patient samples was much greater than between controls and those treated for any given sample. This shows that p53 activation specifically only affects p53 transcriptional target genes, and these represent only a small subset of the large total number of expressed genes. The vast majority of gene transcripts are not affected by MDM2 inhibition, but nevertheless their pattern of expression differs considerably between patient samples.

Alterations in gene expression between RG7388-sensitive and -resistant CLL samples demonstrated significant changes. In total, the expression of 32 transcribed elements, including protein-coding and noncoding transcripts, was significantly upregulated in RG7388-sensitive CLL samples following treatment (fold change ≥ 2 and FDR adjusted *p*-value of less than 0.05) (Figure 2C). The resistant samples’ gene regulation profile showed notable divergence, despite most genes being co-regulated in common with the sensitive ones. After treatment, there was a significant increase in the level of expression of 28 transcribed elements in RG7388-resistant CLL samples (fold change ≥ 2 and FDR adjusted *p*-value < 0.05) (Figure 2D). Genes that revealed statistically significant variations in expression were almost all upregulated.

### 3.3. Differentially Expressed Genes between Control and Treated Samples

**Figure 3:**
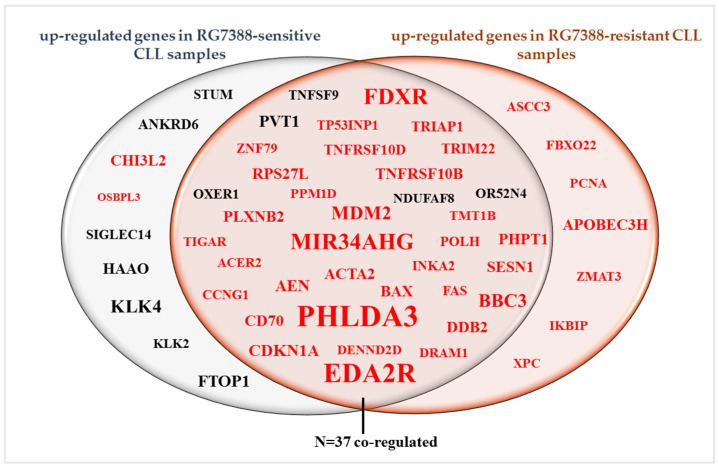
Venn diagram showing upregulated genes that are expressed jointly or only in sensitive or resistant subgroups. Upregulated genes after treatment with 1 µM RG7388 for 6 h in CLL patient samples either sensitive (left) or resistant (right) to RG7388 are shown. Thirty-seven genes were co-regulated in both groups, while seven genes were upregulated only in the resistant CLL cells. Differentially expressed genes had adjusted *p*-values < 0.05. Genes with a low normalized count value (< 50) and an expression fold change lower than 1.5 were excluded. Direct p53 transcriptional targets [10,11] are shown in red. The font size is used to indicate the relative level of fold change.

One of the aims of this RNA-seq study was to identify potential response biomarkers for RG7388-resistant *TP53*^WT^ CLL samples. Following mapping of RNA-seq reads to the genome, the data were converted into a quantitative measure of gene expression represented as the normalized read count. The fold change value, which describes how much a quantity changes from one condition to another, was also calculated. Differentially expressed genes (DEGs) included for the downstream analysis had adjusted *p*-values < 0.05. Genes with a low normalized count value (< 50) were excluded from the downstream analysis (Figure 1A). After normalization and filtering of RNA-seq data, a list of DEGs between 1 µM RG7388 treated and DMSO control CLL patient samples was generated. When the genes with an expression fold change lower than 1.5 were excluded from the analysis, the number of upregulated genes was 46 for the RG7388-sensitive subgroup of patients while it was 44 for the RG7388-resistant subgroup. Supplementary Tables S1 and S2 show the list of upregulated genes with corresponding fold changes and *p*-adj values for the CLL patient samples sensitive or resistant to treatment with RG7388, respectively. Thirty-seven genes were co-regulated in both subgroups, while seven and nine genes were group-specific for the resistant and sensitive subgroups, respectively (Figure 3).

### 3.4. RNA-Seq Analysis Reveals Candidate Genes and Pathways Associated with Resistance to RG7388

To further examine the differentially expressed genes between drug-sensitive and -resistant responders, we performed pathway enrichment analyses on the significantly upregulated gene groups using The Database for Annotation, Visualization and Integrated Discovery (DAVID), which provides an extensive collection of functional annotation tools to assist researchers in exploring the biological relevance of extensive gene lists [12]. The enriched pathways are given along with the *p*-values of their significance. The Benjamini–Hochberg method was used to correct the enrichment *p*-values for multiple testing [13].

From the RNA-seq data, we identified seven upregulated genes that were associated with RG7388 resistance (Figure 3). A core analysis using the pathway analysis tool on these genes revealed the top enriched canonical pathways shown in Table 2. The genes that were co-regulated in both the RG7388-sensitive and -resistant subgroups were mainly related to the regulation of apoptosis and p53 signaling, as expected. The genes that were differentially upregulated only in the resistant CLL cell samples were found to be DNA damage related (Table 2). The pathway analysis for the nine genes that were differentially upregulated only in the sensitive CLL cells revealed no significant associated pathway enrichment (*p*-adj > 0.05).

### 3.5. The RG7388-Resistant Subgroup of CLL Samples Had Lower Basal and Induced Levels of E2F7 Expression Compared to the RG7388-Sensitive Subgroup

**Figure 4:**
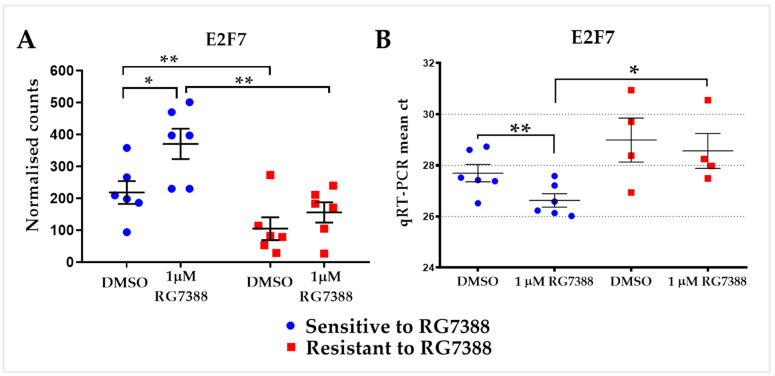
*E2F7* expression in primary CLL cells in response to RG7388. (**A**). Scatter plot showing differential expression of transcription factor gene *E2F7* between p53-functional primary CLL cell samples that are sensitive (*n* = 6) and resistant to RG7388 (*n* = 6) with and without exposure to 1 µM RG7388 for 6 h. The *y*-axis represents normalized RNA-seq gene expression counts. Changes in the normalized count were compared by a multiple *t*-test using the Holm–Sidak method; sensitive vs. resistant DMSO, *p* = 0.001; sensitive vs. resistant 1 µM RG7388, *p* = 0.004. Statistically significant differences (* *p* < 0.05; ** *p* < 0.01) are indicated. (**B**). Scatter plot shows Ct values (cycle number to reach critical threshold) for E2F7 measured by qRT-PCR to validate RNA-seq results using an independent set of primary CLL samples. *TP53*^WT^ CLL samples that are sensitive (*n* = 6) or resistant (*n* = 4) to RG7388 were exposed to 1 µM RG7388 for 6 h. Change in ct values were compared by an unpaired *t*-test; sensitive vs. resistant 1 µM RG7388, *p* = 0.015. Paired *t*-test was applied for sensitive samples DMSO vs. 1 µM RG7388, *p* = 0.0013. Statistically significant differences (* *p* < 0.05; ** *p* < 0.01) are indicated. NB: Lower ct value indicates higher expression.

Regarding basal gene expression levels, the only genes showing a statistically significant difference between the subgroups (multiple *t*-test using Holm–Sidak method for each gene, *p* > 0.05; Supplementary Table S3) were E2F7, USP14, and YIPF4. The noteworthy finding was that RG7388-resistant samples had significantly lower basal and induced expression of the transcription factor gene *E2F7* than did sensitive responders based on the data from RNA-seq (Figure 4A). To confirm the differential expression of *E2F7* between RG7388-sensitive and -resistant CLL samples, qRT-PCR was performed with a different subset of p53-functional primary CLL samples following treatment with 1 µM RG7388 for 6 h. The qRT-PCR Ct results confirmed the lower expression of *E2F7* (Figure 4B).

### 3.6. Validation of RNA-Seq Results Using Real-Time PCR

**Figure 5:**
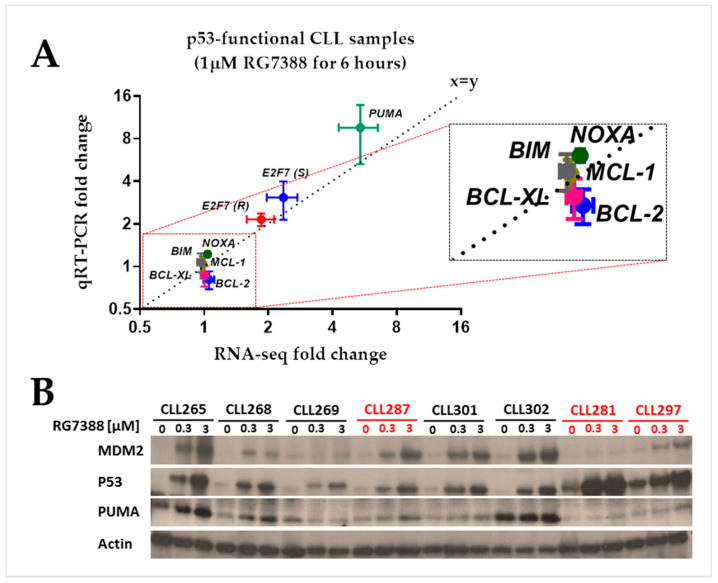
(**A**). Correlation between RNA-seq and qRT-PCR. Correlation plots indicating the relationship between qRT-PCR results (fold change; *y*-axis, *n* = 7 samples) for 7 selected genes and the corresponding data from RNA-seq analysis (fold change; *x*-axis, *n* = 12 samples). RNA samples were extracted separately for RNA-seq and qRT-PCR. E2F7 expression change is shown separately for the primary CLL samples that are sensitive (S) or resistant (R) to RG7388. A statistically significant Pearson correlation was found between the expression levels measured using qPCR and RNA-seq (*p* < 0.0001). Bars represent standard error. (**B**). Western blot analysis showing representative examples of concentration-dependent PUMA protein induction by ex vivo treatment of CLL samples with RG7388 for 24 h (CLL265 and CLL268). Two examples of non-functional p53 stabilization with minimal downstream PUMA expression are included (CLL281 and CLL297) together with one example of high basal PUMA expression, which is further increased by RG7388 (CLL302). RG7388-resistant CLL samples are indicated in red.

MDM2 inhibitors, including RG7388, selectively trigger pro-apoptotic genes and apoptosis in CLL cells but not in normal B cells [2]. The consequence of changes in pro-apoptotic genes also requires consideration of the status of anti-apoptotic gene expression and function. Differential regulation of MCL-1 in human primary and drug-resistant cancer cells including CLL [18] has been reported to play a critical role in the regulation of apoptosis [19]. To investigate the potential involvement of anti-apoptotic factors in the differential response to MDM2 inhibitors in p53 wild-type cells, the basal expression of individual anti-apoptotic BCL-2 family members was assessed using the qRT-PCR method. This was also performed for the purpose of validating the RNA-seq results. A panel of anti-apoptotic genes (*BCL2*, *MCL1*, and *BCL2L1* (BCL-XL)), and the pro-apoptotic genes *PMAIP1* (NOXA) and *BCL2L11* (BIM), were analyzed by independent qRT-PCR in a subset of primary CLL samples. This additional analysis showed no significant changes in mRNA expression when compared with the large change in *PUMA* mRNA (Figure 5A). A statistically significant Pearson correlation was found between the expression levels measured using qRT-PCR and RNA-seq (*p* < 0.0001). Consistent with transcript analysis, Western immunoblotting verified that CLL samples exhibited a PUMA protein increase following RG7388 treatments (Figure 5B).

## 4. Discussion

RNA-seq is a widely used technique across biological disciplines that enables a comprehensive transcriptome analysis in cell lines or clinical samples. One of the most common uses of the RNA-seq approach is to find differences in gene expression between two or more conditions. The main objective in this study was to determine how the gene expression profile in CLL primary cells changes after treatment with one of the second-generation MDM2–p53-binding antagonists RG7388 (idasanutlin). RG7388 was the first MDM2–p53-binding antagonist to progress through phase II clinical studies (ClinicalTrials.gov Identifier: NCT02633059), and according to preliminary clinical trial outcomes, shows encouraging potential for inclusion as a therapeutic drug for wild-type p53 leukemia [3].

Our earlier research explored mRNA profiles of CLL samples at different time points, and we concluded that treatment with 1 µM RG7388 for 6 h was the optimum approach to detect the transcriptional induction of several p53 pathway genes at a time and dose well before the consequent cytotoxic effects and secondary changes in CLL cells become evident [2]. Therefore, 6 h treatment of CLL cells with 1 µM RG7388 was chosen for the RNA-seq analysis of direct transcriptional changes. As anticipated, the RNA-seq analysis showed significant changes in the expression of a number of target genes associated with p53 signaling, as described in the meta-analyses review from 16 genome-wide datasets taken as a reference [10]. However, interestingly, there were also several upregulated genes that had not previously been described and may be novel context-specific p53-regulated genes in CLL, which are worthy of further exploration. These included *KLK4*, *OXER1*, *FTOP1,* and *OR52N4* (genes with >2 fold change). Kallikreins are a subgroup of serine proteases with diverse physiological functions [20]. *KLK4* (Kallikrein-related peptidase *4*) is considered to be an oncogene associated with various types of cancers. Elevated *KLK4* expression has been shown to be associated with poor prognosis in breast cancer [21] and ovarian cancer [22]. The *OR52N4* gene encodes olfactory receptor 52N4, which interacts with odorant molecules in the nose, to initiate a neuronal response that triggers the perception of a smell [23]. Since a direct link cannot be established between the function of this protein and the pathway targeted by RG7388, further investigation is required. There was also an increase in the expression of one of the established p53-target genes *PVT1* [24], which had not previously been reported in CLL. The *PVT1* gene has been identified as an oncogene encoding PVT1 long noncoding RNA, and increased expression of *PVT1* has been found to be associated with numerous cancer types (reviewed in [25]). In our study, microRNA-34a (miR-34a; encoded by the *MIR34AHG* gene), a critical direct transcriptional target of p53, was among the most highly upregulated genes. Characterization of the miR-34a primary transcript and promoter has shown that this miRNA is directly transactivated by p53 [11]. MiR-34a levels were also reported to be upregulated after DNA damage in the presence of functional p53 in CLL [26]. The high induction of its expression by RG7388 is consistent with functional p53 activation.

**Figure 6:**
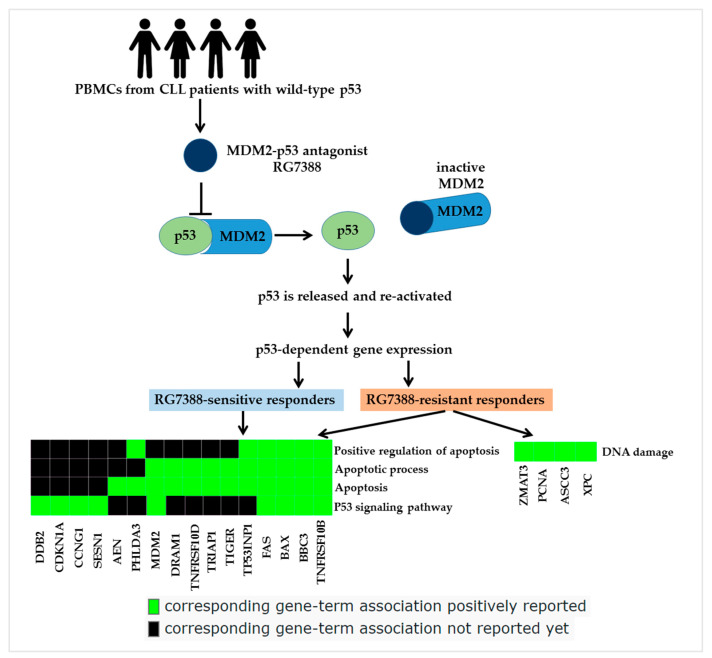
Regulation of different cellular events in RG7388-resistant CLL samples. Binding of MDM2–p53 antagonist RG7388 to the p53-binding pocket of MDM2 releases and activates wild-type p53. Increased p53 activity leads to upregulation of pro-apoptosis pathway genes. However, in RG7388-resistant samples, DNA damage response pathway genes are also upregulated in addition to the co-regulated genes.

The DAVID bioinformatics web tool was utilized to conduct pathway enrichment analyses in order to look into the genes that were expressed differentially between drug-sensitive and -resistant responders. The genes that were differentially expressed solely in the resistant samples were linked with the DNA damage response pathway, whereas the genes that were co-upregulated in both RG7388-sensitive and -resistant patient samples were primarily connected to the regulation of apoptosis and p53 signaling (Table 2; Figure 6). Prior research has addressed questions about how to best to combine MDM2 inhibitors (MDM2i) with other therapies in patients with wild-type p53 cancers. In one approach, clinical trials have investigated MDM2i in combination with DNA-damaging drugs, since DNA-damaging chemotherapy remains the standard of care for many malignancies [27].

MDM2i can potentially protect wild-type p53 cancer cells and normal tissue cells from drug-induced DNA damage. It is suggested to do so by increasing the levels of the cyclin-dependent kinase inhibitor p21 protein, which induces G1 cell cycle checkpoint arrest and prevents cells from replicating DNA on a damaged template, allowing time for DNA repair. Cells deficient in p21 were found to have impaired p53-dependent cell cycle arrest, which prevented them from arresting following DNA damage [28]. p21 is critical for cancer cell survival and resistance to treatments that damage DNA. The loss of p21 reduces the protective effect of MDM2 inhibitors on DNA and boosts the effectiveness of MDM2 and DNA-damaging agents when combined. Furthermore, in a previous study by our group, we showed that p21 is vulnerable to inhibition with a spliceosome inhibitor E7107, and a novel p21 aberrant isoform with a compromised cyclin-dependent kinase inhibitory activity is generated [6]. The protective effect of normal p21-mediated growth inhibition is lost with the switch to the aberrant p21 isoform, which is unable to localize to the nucleus, thus sensitizing E7107-treated cells to the MDM2 inhibitor RG7388. The upregulation of DNA damage pathway gene expression shown in the RNA-seq data for RG7388-resistant samples suggests combining MDM2 inhibitors with DNA-damaging agents needs careful consideration of appropriate biomarkers for patient selection and treatment scheduling. The RNA-Seq data show clear evidence of p53-dependent pro-apoptotic gene induction and support a strategy of enhancing CLL cell apoptosis by combining MDM2 inhibitors with BCL-family inhibitors such as venetoclax and navitoclax, as is currently being explored in other leukemia clinical trials [3].

Regarding basal gene expression levels, there was no statistically significant distinction between the subgroups (Supplementary Table S3) except for E2F7, USP14, and YIPF4. The basal expression of the transcription factor gene *E2F7* was found to be significantly lower in RG7388-resistant samples when compared with sensitive responders. The QRT-PCR results with an independent set of samples confirmed the differential expression of *E2F7* (Figure 4). *E2F7* has previously been described as a p53 target [10,29]. It plays an essential role in the regulation of cell cycle progression [30]. The p53-dependent transcriptional upregulation of *E2F7* directly represses important players in the DNA damage response such as *RAD51*, *CHEK1,* and *BRCA1* [29,31,32]. E2F7 was identified as a key senescence regulator with tumor-suppressive activity that provides a link between the RB and p53 pathways during cellular senescence [33]. The overexpression of *E2F7* was shown to block S-phase entry [31,34]. Taken together, reduced *E2F7* expression might play a role in resistance to RG7388.

The loss of p53 or suppression of p53 by its primary negative regulator MDM2 is a common feature of human cancers, which leads to defective cell cycle checkpoint control and reduced induction of apoptosis. Interruption of the binding between p53 and the E3-ubiquitin ligase MDM2, to prevent ubiquitination-mediated degradation of p53, results in p53 accumulation and activation of the growth-inhibitory and proapoptotic functions of p53 [35]. Another notable finding from our RNA sequencing study was the low basal level of USP14 in RG7388-resistant cells (Supplementary Table S3). Ubiquitin-specific peptidase 14 (USP14) is a deubiquitinase closely related to the human 19S proteasome [36]. It is highly expressed in a variety of cancers including lung carcinoma and leukemia, and it has been reported to be associated with a poor prognosis [37,38,39]. RG7388-resistant CLL cells may benefit from the differential basal expression of USP14, which controls the fate of p53 through proteasomal degradation by removing ubiquitin chains. Therefore, activating USP14 may abolish the protective effect of low expression on RG7388-resistant cells and offer a new combined treatment strategy.

RG7388 selectively triggers pro-apoptotic genes and apoptosis in CLL cells but not in normal B cells [2]. In order to expand the gene panel in our previous study, as well as validate the RNA-seq results, the expression of some of the anti-apoptotic BCL-2 family members and pro-apoptotic genes was assessed using the qPCR method. The anti-apoptotic gene *BCL-2* has been shown to be overexpressed in CLL and inhibits the activity of pro-apoptotic BH3-only family members, such as PUMA [40,41,42]. In addition, differential regulation of another anti-apoptotic *BCL-2* family member *MCL-1*, which plays a critical role in the regulation of apoptosis, has been reported in human primary and drug-resistant cancer cells, including CLL [18,19]. Additional qRT-PCR analysis showed no significant changes in mRNA expression following treatment with RG7388, compared to the large change in *PUMA* mRNA. However, the basal and drug-induced mRNA levels of anti-apoptotic genes were considerably higher than those of *PUMA*, suggesting a high expression of anti-apoptotic genes in these CLL cells consistent with the literature. Our previously reported marked increase in *PUMA* expression was confirmed in the present study using a different set of CLL primary samples by both RNA-seq and qRT-PCR. Parallel to the other methods, Western blot analysis verified that CLL samples exhibited a PUMA protein increase following RG7388 treatments.

## 5. Conclusions

The highly heterogeneous genetic nature of CLL and the development of resistance to current treatments require new strategies for the efficient long-term management of CLL patients. The main purpose of this study was to contribute to these strategies. Novel biomarkers that predict and potentially dictate the cellular response of CLL cells to MDM2 inhibition were identified. This research provides preclinical data to guide the exploration of drug combination strategies with MDM2 inhibitors, supporting the conduct of future clinical trials and the development of associated biomarkers to improve the outcomes for CLL patients.

## Figures and Tables

**Table 1:** Primers used for qRT-PCR.

Genes	Primer Forward (5′-3′)	Primer Reverse (5′-3′)
GAPDH	CGACCACTTTGTCAAGCTCA	GGGTCTTACTCCTTGGAGGC
PUMA (BBC3)	ACCTCAACGCACAGTACGA	CTGGGTAAGGGCAGGAGTC
BCL2	GGTGGGGTCATGTGTGTGG	CGGTTCAGGTACTCAGTCATCC
MCL1	GTGCCTTTGTGGCTAAACACT	AGTCCCGTTTTGTCCTTACGA
BCL2L1 (BCL-XL)	QIAGEN QUANTITECT PRIMER ASSAY QT00236712	
PMAIP1 (NOXA)	TGCTACACAATGTGGCGTC	ACTTGGACATGGCCTCCCTTA
^1^ BCL2L11 (BIM)	TAAGTTCTGAGTGTGACCGAGA	GCTCTGTCTGTAGGGAGGTAGG
E2F7	ATATCTTTGTGTGCAGTCTCCTG	AAGACGGCAGCTGACCTGA

^1^ BCL2L11 (BIM) primers amplify exon-2 and detect all of the splice variants that have been described (BimEL, BimL, BimS, and BimAD) [8].

**Table 2:** Summary of DAVID pathway enrichment analysis.

DEGs	Subgroup	Annotation Cluster	Enriched Pathway	Count	*p*-Value	*p*-adj (Benjamini–Hochberg)
Up	Upregulated genes only in resistant CLLs (*n* = 7) Enrichment score: 2.04	^1^ UniProt	DNA damage	4	5.0 × 10^−4^	7.5 × 10^−3^
	Co-upregulated genes in both sensitive and resistant CLLs (*n* = 37) Enrichment score: 6.14	UniProt	Apoptosis	12	2.5 × 10^−9^	6.9 × 10^−8^
		^2^ QuickGO	Apoptotic process	10	6.2 × 10^−7^	1.3 × 10^−4^
		QuickGO	Positive regulation of apoptotic process	6	2.4 × 10^−4^	2.5 × 10^−2^
		^3^ KEGG	P53 signaling pathway	9	3.8 × 10^−12^	3.8 × 10^−10^

^1^ UniProt: The UniProt Knowledgebase [14]; ^2^ QuickGO: The Gene Ontology (GO) [15,16]; ^3^ KEGG: Kyoto Encyclopedia of Genes and Genomes [17]. DEGs: differentially expressed genes.

## Data Availability

The data presented in this study and released under a CC-BY 4.0 license are available upon request from the corresponding authors.

## Supplementary Materials

The following supporting information can be downloaded at https://www.mdpi.com/article/10.3390/biomedicines12071388/s1, Figure S1: Agilent Bioanalyzer electropherogram with RNA peaks and gel representation; Table S1: Up-regulated genes in CLL primary samples sensitive to RG7388 (comparison between 1 μM RG7388 treated vs. DMSO control); Table S2: Up-regulated genes in CLL primary samples resistant to RG7388 (comparison between 1 μM RG7388 treated vs. DMSO control); Table S3: Comparison of basal expression between RG7388-sensitive and -resistant subgroups of CLL primary samples for the top seventeen genes based on their *p*-adj values; Table S4: RNA concentrations and quality.
