# Supplementary material for: RNA Sequencing Reveals Candidate Genes and Pathways Associated with Resistance to MDM2 Antagonist Idasanutlin in TP53 Wild-Type Chronic Lymphocytic Leukemia
Source: Biomedicines. 2024 Jun 22;12(7):1388. doi: 10.3390/biomedicines12071388 (PMC11274024; doi:10.3390/biomedicines12071388)
Supplement: Supplementary file 1 [file biomedicines-12-01388-s001.zip › biomedicines-3043217-supplementary.pdf]

Supplementary Figure S1

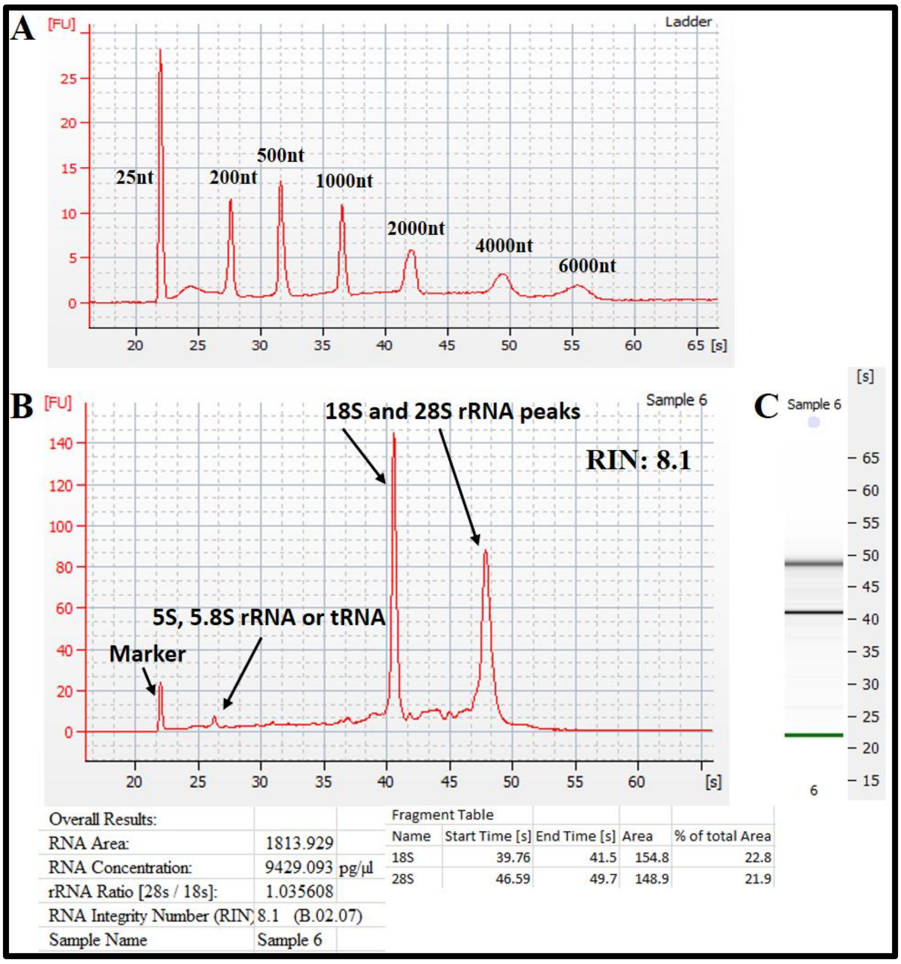

Supplementary Figure S1. Agilent Bioanalyzer electropherogram with RNA peaks and gel representation. A. Electropherogram showing the reference RNA ladder. **B.** RNA concentration and RIN (RNA Integrity Number) value are calculated and presented for a representative RNA sample. The ratio of 28S:18S rRNA molecules are calculated based on the area under each curve. Peaks associated with other rRNA or tRNA molecules has been arrowed in addition to the control molecule (marker). **C.** A total RNA gel like-image (on the right) produced by the Bioanalyzer. The 28S and 18S distinctive ribosomal RNA bands are observed.

Supplementary Table S1. Up-regulated genes in CLL primary samples sensitive to RG7388 (comparison between 1 µM RG7388 treated vs. DMSO control).

Gene	Description	[1]FC	*p*-Adj	[2]RS
PHLDA3	Pleckstrin homology like domain family A member 3	11.6	5.94E-08	9
EDA2R	Ectodysplasin A2 receptor	7.6	7.00E-08	7
FDXR	Ferredoxin reductase	6.9	4.51E-10	7
MIR34AHG	MIR34A host gene	6.9	2.46E-25	0
BBC3	BCL2 binding component 3	3.8	0.0003	7
MDM2	MDM2 proto-oncogene	3.7	2.29E-06	15
KLK4	Kallikrein related peptidase 4	3.2	0.00031	0
ACTA2	Actin, alpha 2, smooth muscle, aorta	3.1	1.94E-12	6
PLXNB2	Plexin B2	3	1.03E-23	4
PVT1	Pvt1 oncogene (non-protein coding)	3	0.00089	0
CDKN1A	Cyclin dependent kinase inhibitor 1A	2.8	0.00595	16
PHPT1	Phosphohistidine phosphatase 1	2.8	1.00E-10	5
AEN	Apoptosis enhancing nuclease	2.8	1.04E-05	10
OXER1	Oxoeicosanoid receptor 1	2.7	6.03E-06	0
CD70	CD70 molecule	2.7	6.34E-20	1
SESN1	Sestrin 1	2.6	2.64E-16	10
DDB2	Damage specific DNA binding protein 2	2.5	1.0E-10	13
TRIM22	Tripartite motif containing 22	2.5	7.05E-18	9
FTOP1	Fat mass and obesity associated pseudogene 1	2.5	0.0077	0
TNFRSF10B	TNF receptor superfamily member 10b	2.5	3.76E-05	1
BAX	BCL2 associated X, apoptosis regulator	2.4	1.11E-14	11
TRIAP1	TP53 regulated inhibitor of apoptosis 1	2.3	6.83E-12	12
RPS27L	Ribosomal protein S27 like	2.2	1.45E-09	12
OR52N4	Olfactory receptor family 52 subfamily N member 4	2.2	6.41E-05	0
FAS	Fas cell surface death receptor	2	2.63E-07	10
TP53INP1	Tumour protein p53 inducible nuclear protein 1	2	3.26E-16	10

HAAO	3-hydroxyanthranilate 3,4-dioxygenase	2	0.029	0
CHI3L2	Chitinase 3 like 2	2	1.37E-07	1
ANKRD6	Ankyrin repeat domain 6	1.9	7.98E-05	0
TNFRSF10D	TNF receptor superfamily member 10d	1.9	4.18E-06	6
POLH	DNA polymerase eta	1.8	0.0019	11
PRDM1	PR/SET domain 1	1.8	0.03	6
STUM	Mechanosensory transduction mediator homolog	1.8	0.02	0
SIGLEC14	Sialic acid binding Ig like lectin 14	1.8	0.00027	0
TNFSF9	TNF superfamily member 9	1.8	0.00119	0
TMT1A	Methyltransferase like 7A	1.7	3.34E-10	1
ACER2	Alkaline ceramidase 2	1.7	0.00067	7
CCNG1	Cyclin G1	1.7	5.34E-25	9
ZNF79	zinc finger protein 79	1.7	4,89E-07	8
TIGAR	TP53 induced glycolysis regulatory phosphatase	1.7	2,23E-07	14
INKA2	family with sequence similarity 212 member B	1.7	0,006	8
KLK2	kallikrein related peptidase 2	1.6	0,0093	0
DRAM1	DNA damage regulated autophagy modulator 1	1.6	0,0039	10
DENND2D	DENN domain containing 2D	1.5	6,7E-07	2
OSBPL3	oxysterol binding protein like 3	1.5	0,0000078	2
NDUFAF8	NADH:ubiquinone oxidoreductase complex assembly factor 8	1.5	0,0031	0

[1]**FC**: Fold Change. [2]**RS**: Regulation Score. The number of genome-wide data sets (out of 16) in which the corresponding gene has been identified as an activated p53 target (Fischer 2017).

Supplementary Table S2. Up-regulated genes in CLL primary samples resistant to RG7388 (comparison between 1 μM RG7388 treated vs. DMSO control).

Gene	Description	[1]FC	*p*-Adj	[2]RS
PHLDA3	pleckstrin homology like domain family A member 3	11.7	2.28E-08	9
EDA2R	ectodysplasin A2 receptor	7.5	1.02E-08	7
MIR34AHG	MIR34A host gene	6.5	2.50E-23	0
FDXR	ferredoxin reductase	6.4	2.82E-09	7
BBC3	BCL2 binding component 3	5.2	4.12E-07	7
MDM2	MDM2 proto-oncogene	5.0	5.56E-10	15
AEN	apoptosis enhancing nuclease	3.5	2.78E-09	10
CDKN1A	cyclin dependent kinase inhibitor 1A	3.1	0.00084	16
PVT1	Pvt1 oncogene (non-protein coding)	2.9	0.0014	0
PHPT1	phosphohistidine phosphatase 1	2.8	4.68E-11	5
SESN1	sestrin 1	2.8	1.17E-18	10
PLXNB2	plexin B2	2.7	1.17E-18	4
ACTA2	actin, alpha 2, smooth muscle, aorta	2.6	1.29E-08	6
RPS27L	ribosomal protein S27 like	2.6	5.14E-14	12
TNFRSF10B	TNF receptor superfamily member 10b	2.6	6.48E-06	1
CD70	CD70 molecule	2.6	1.17E-18	1
DDB2	damage specific DNA binding protein 2	2.5	3.59E-11	13
BAX	BCL2 associated X, apoptosis regulator	2.5	3.16E-16	11
APOBEC3H	apolipoprotein B mRNA editing enzyme catalytic subunit 3H	2.5	0.00026	5
TRIAP1	TP53 regulated inhibitor of apoptosis 1	2.3	2.69E-12	12
TRIM22	tripartite motif containing 22	2.2	5.27E-13	9
TNFRSF10D	TNF receptor superfamily member 10d	2.1	1.53E-08	6
OXER1	oxoeicosanoid receptor 1	1.9	0.016	0
TP53INP1	tumour protein p53 inducible nuclear protein 1	1.9	2.62E-13	10
CCNG1	cyclin G1	1.9	8.42E-38	9
POLH	DNA polymerase eta	1.9	0.00042	11
OR52N4	olfactory receptor family 52 subfamily N member 4 (gene/pseudogene)	1.9	0.0057	0

INKA2	family with sequence similarity 212 member B	1.9	5.18E-05	8
ASCC3	activating signal cointegrator 1 complex subunit 3	1.8	1.61E-08	8
FAS	Fas cell surface death receptor	1.8	0.00033	10
ZMAT3	zinc finger matrin-type 3	1.7	7.85E-06	12
TNFSF9	TNF superfamily member 9	1.7	0.0032	0
XPC	XPC complex subunit, DNA damage recognition and repair factor	1.7	5.14E-14	11
FBXO22	F-box protein 22	1.7	0.0041	9
ZNF79	zinc finger protein 79	1.7	4.98E-07	7
TMT1B	methyltransferase like 7A	1.6	4.04E-09	1
PCNA	proliferating cell nuclear antigen	1.6	0.00011	5
DRAM1	DNA damage regulated autophagy modulator1	1.6	0.0016	9
IKBIP	IKBKB interacting protein	1.6	1,07E-07	6
ACER2	alkaline ceramidase 2	1.5	0,049	7
PPM1D	protein phosphatase, Mg2+/Mn2+ dependent 1D	1.5	0,00039	12
DENND2D	DENN domain containing 2D	1.5	0,000011	2
TIGAR	TP53 induced glycolysis regulatory phosphatase	1.5	0,001	14
NDUFAF8	NADH:ubiquinone oxidoreductase complex assembly factor 8	1.5	0,006	0

[1]**FC**: Fold Change. [2]**RS**: Regulation Score. The number of genome-wide data sets (out of 16) in which the corresponding gene has been identified as an activated p53 target (Fischer 2017).

Supplementary Table S3. Comparison of basal expression between RG7388-sensitive and -resistant subgroups of CLL primary samples for the top seventeen genes based on their *p*-adj values.

Gene	Description	[1]*p*-adj	[2]FC
E2F7	**E2F transcription factor 7**	**0.0014**	**3.1**
USP14	ubiquitin specific peptidase 14	0.0228	1.6
YIPF4	Yip1 domain family member 4	0.0263	1.5
ANKRD6	ankyrin repeat domain 6	0.0684	0.6
NRCAM	neuronal cell adhesion molecule	0.0685	5.9
PAIP1	poly(A) binding protein interacting protein 1	0.0904	1.2
C1RL-AS1	C1RL antisense RNA 1	0.1297	0.8
NOS3	nitric oxide synthase 3	0.1626	0.3
CEP170B	centrosomal protein 170B	0.2223	5.1
CC2D1B	coiled-coil and C2 domain containing 1B	0.2688	0.9
ZNF778	zinc finger protein 778	0.2963	0.8
TSPAN32	tetraspanin 32	0.3038	0.3
TICAM2	toll like receptor adaptor molecule 2	0.4325	0.4
GOLGA8G	golgin A8 family member G	0.4537	1.5
TBC1D27P	TBC1 domain family member 27, pseudogene	0.5807	0.7
SPEN	spen family transcriptional repressor	0.6481	1.1
SH3BP2	SH3 domain binding protein 2	0.9147	1.0

[1]*p*-adj values of multiple t-test using Holm-Sidak method. [2]FC: fold change. The ratio of basal gene expression between RG7388-sensitive (n=6) and resistant (n=6) subgroups of p53-functional primary CLL samples (sensitive/resistant).

Supplementary Table S4. RNA concentrations and quality.

Tumour ID	RG7388 LC_{50} (μM)	Treatment (1 μM RG7388)	Nanodrop			Bioanalyzer	
			conc. (ng/μl)	260/280	260/230	RIN	conc. (ng/μl)
**276**	**1.7**	-	17.5	2.04	0.58	**8.2**	19.6
		+	86.9	1.88	1.04	**8.3**	14.4
**277**	**>3**	-	74.2	2.03	0.94	**8.8**	97.5
		+	81.6	2.07	2.11	**8.5**	117.7
**279**	**2.37**	-	93.5	1.84	1.53	**6.5**	31.0
		+	93.8	1.84	1.88	**6**	13.7
**288**	**>10**	-	47.6	2.03	1.4	**7.1**	23.9
		+	49.3	2.07	1.58	**7.1**	17.3
**290**	**>10**	-	87.2	2.06	1.46	**7.7**	38.2
		+	78.2	2.06	1.14	**7.4**	34.3
**296**	**2.3**	-	167.4	2.05	2.12	**8.8**	203.9
		+	143.7	2.03	2.25	**8.4**	210.7
**265**	**0.28**	-	22.9	2.06	1.27	**7.8**	30.6
		+	27.2	2.10	1.75	**7.9**	20.8
**278**	**0.19**	-	38.0	1.91	1.19	**8.1**	26.1
		+	22.4	1.95	1.37	**7.7**	28.3
**282**	**0.63**	-	30.0	1.90	1.36	**7.2**	23.5
		+	50.2	2.09	1.98	**7.5**	25.9
**284**	**0.56**	-	92.4	2.08	2.14	**8.7**	135.5
		+	90.0	2.04	1.85	**8.7**	138.0
**285**	**0.34**	-	34.6	2.03	0.46	**8.1**	16.3
		+	32.6	2.03	1.07	**7.3**	18.5
**289**	**0.65**	-	123.9	2.03	2.37	**8.5**	206.2
		+	112.2	2.04	2.18	**7.8**	182.8
